# Current Evidence on the Role of Pediatric Dentists in the Multidisciplinary Management of Pediatric Obstructive Sleep Apnea

**DOI:** 10.3390/diagnostics16060843

**Published:** 2026-03-12

**Authors:** Antonino Lo Giudice, Alessia Malgioglio, Antonino Maniaci, Ignazio La Mantia, Alberto Bianchi, Salvatore Cocuzza

**Affiliations:** 1 Department of Medical-Surgical Specialties, School of Dentistry, Section of Pediatric Dentistry, University of Catania, Via Santa Sofia 78, 95123 Catania, Italy; 2 Department of Medicine and Surgery, University of Enna Kore, 94100 Enna, Italy; 3 Department of Medical, Surgical and Technological Sciences, Section of Otolaryngology, University of Catania, 95123 Catania, Italy; igolama@gmail.com (I.L.M.);; 4 Department of Medical-Surgical Specialties, School of Dentistry, Section of Maxillo-Facial Surgery, University of Catania, Via Santa Sofia 78, 95123 Catania, Italy

**Keywords:** OSAS, children, pediatric dentistry, orthodontics

## Abstract

Pediatric obstructive sleep apnea (OSA) is a prevalent and underdiagnosed condition associated with significant neurocognitive, behavioral, and systemic consequences. Sleep-related breathing disorders (SRBDs) in children range from primary snoring to OSA, with even mild forms increasingly linked to adverse outcomes. Given their frequent contact with pediatric patients, pediatric dentists and orthodontists are uniquely positioned to contribute to early identification and management within a multidisciplinary framework. Objectives: This narrative review aimed to summarize and critically appraise current evidence to clarify the clinical role, scope of practice, and responsibilities of pediatric dentists and orthodontists within the multidisciplinary management of pediatric obstructive sleep apnea. A comprehensive literature search was conducted in PubMed, Scopus, Web of Science, and EMBASE up to 1 November 2025. Review articles addressing the involvement of pediatric dentists and orthodontists in pediatric OSA were included. No restriction was applied to language or publication year. Two authors independently performed study selection and data extraction. The methodological quality and data extraction of the studies were structured according to the SANRA scale. Ten studies were deemed suitable for inclusion in the current review. After examination of the full texts, the available evidence was filtered into specific clinical domains aimed at clarifying the role of the pediatric dentist and orthodontist in the management of pediatric obstructive sleep apnea (OSA). Qualitative thematic analysis of the included studies identified three main areas in which pediatric dentists and orthodontists contribute to the management of pediatric OSA. The first area involves screening through recognition of clinical signs and symptoms, use of validated questionnaires, and identification of craniofacial and occlusal features associated with increased airway risk. The second area concerns participation in the diagnostic–therapeutic pathway and multidisciplinary care, including timely referral, clinical documentation, and collaboration with pediatricians, otolaryngologists, and sleep specialists. The third area relates to orthodontic treatments such as rapid maxillary expansion and mandibular advancement appliances, which may provide adjunctive benefits in selected patients, although current evidence is limited by heterogeneity and growth-related confounding factors. Pediatric dentists and orthodontists play a pivotal yet complementary role in the management of pediatric OSA. In particular, all the involved specialists are encouraged to actively participate in the screening process, interdisciplinary communication, and diagnostic and therapeutic decision-making processes.

## 1. Introduction

Sleep is a fundamental physiological process that is essential for normal growth and development in children. Beyond its role in somatic growth, sleep plays a crucial role in immune function, neurocognitive maturation, and emotional regulation. Chronic sleep disruption during childhood has been associated with obesity, impaired cognitive performance, behavioral disturbances, and reduced quality of life [1]. Despite these well-documented consequences, awareness of pediatric sleep disorders remains limited. Epidemiological data indicate that approximately 25–40% of children experience some form of sleep disturbance, including behavioral, neurological, and respiratory-related conditions, with relevant health and social implications [2].

Within this spectrum, sleep-related breathing disorders (SRBDs) comprise a heterogeneous group of conditions characterized by partial or complete upper airway obstruction during sleep, resulting in impaired ventilation [3]. These disorders alter sleep architecture, leading to fragmented sleep and reduced sleep quality. Consequently, affected children may present with excessive daytime sleepiness, behavioral dysregulation, and an increased risk of systemic complications, including cardiovascular dysfunction and growth impairment [4,5,6,7]. SRBDs range from primary snoring (PS) to obstructive sleep apnea (OSA). OSA is defined by recurrent episodes of upper airway obstruction during sleep, frequently associated with oxygen desaturation and/or sleep arousals [8]. In children, the pathophysiology of OSA involves sleep-related hypotonia of the pharyngeal dilator muscles, which may be exacerbated by anatomical and functional factors, such as reduced oropharyngeal airway dimensions, posterior tongue position, increased nasal airway resistance, and dynamic upper airway collapse [9]. The prevalence of pediatric OSA is estimated at 1–4% in the general population and increases to 10–20% among habitually snoring children [10]. Clinical manifestations commonly include habitual snoring, mouth breathing, enuresis, morning headaches, and behavioral symptoms, particularly hyperactivity and inattention [11]. Although primary snoring represents the mildest form within the SRBD spectrum, emerging evidence suggests that even habitual snoring may be associated with subtle neurocognitive and behavioral alterations and a higher prevalence of recurrent otitis media [12].

The diagnosis of pediatric OSA is established by supervised in-laboratory polysomnography (PSG). In children, an apnea–hypopnea index (AHI) ≥ 1 event per hour is generally considered diagnostic. Disease severity is classified as mild (AHI ≥ 1 to <5 events/hour), moderate (AHI ≥ 5 to <10 events/hour), and severe (AHI ≥ 10 events/hour), reflecting pediatric-specific thresholds that differ from adult criteria because of the greater clinical relevance of lower event indices in childhood [8,13]. Although PSG remains the gold standard, its routine use as a screening tool is limited by cost, accessibility, and logistical challenges related to overnight testing in young patients [14]. Therefore, careful clinical history, targeted physical examination, and validated screening questionnaires are recommended to identify children at risk, in accordance with the guidelines of the American Academy of Pediatrics (AAP) [15].

In this context, the management of pediatric OSA requires a coordinated multidisciplinary approach involving pediatricians, otolaryngologists, sleep medicine specialists, and dental professionals. The American Academy of Sleep Medicine (AASM) and the American Academy of Pediatric Dentistry (AAPD) explicitly recognize pediatric dentists and orthodontists as important contributors to the identification and management of sleep-disordered breathing in children. Because dental professionals maintain regular and longitudinal contact with pediatric patients during routine preventive and orthodontic visits, they are uniquely positioned to detect early clinical signs suggestive of OSA. Timely referral to appropriate medical specialists may facilitate earlier diagnosis and intervention, thereby reducing the risk of long-term adverse sequelae. In addition, pediatric dentists and orthodontists may also contribute to the therapeutic management of pediatric OSA in the presence of orthodontic indications. Accordingly, the primary objective of this narrative review is to critically analyze and delineate the complementary role of pediatric dentists and orthodontists within the multidisciplinary management of pediatric obstructive sleep apnea. Specifically, the review focuses on three clinical domains derived from the current evidence: (1) structured screening and risk stratification during routine dental assessment; (2) integration within the diagnostic–therapeutic pathway through coordinated referral and interprofessional collaboration; and (3) evidence-based adjunctive orthodontic interventions in growing patients with confirmed OSA. By clearly defining these domains, this review aims to clarify the scope of dental practice within an established medical diagnostic framework and to provide clinically oriented guidance for pediatric dental professionals.

## 2. Materials and Methods

The present narrative review was designed to summarize and critically appraise the current scientific evidence regarding the role of pediatric dentists and orthodontists in the clinical management of children with obstructive sleep apnea (OSA). A comprehensive literature search strategy was developed using predefined keywords and free-text terms and was applied to studies published up to 1 November 2025. The search strategy included the following terms: sleep apnoea syndrome, upper airway resistance sleep apnoea syndrome, OSA, OSAS, snoring, primary snoring, children, growing subjects, dentist, pediatric dentist, pedodontist, orthodontist, orthodontics, and orthodontic treatment, combined using Boolean operators. The search strategy was adapted for each database and applied to PubMed (MEDLINE), Web of Science, and Scopus (Table 1). These databases were selected because of their extensive coverage of biomedical and dental literature, ensuring high relevance to the topic of pediatric OSA. The literature search was conducted without restrictions on language, year of publication, or publication status. In addition, manual searching of the reference lists of all included studies was performed to identify potentially relevant articles. Duplicate records were removed prior to screening. Two reviewers (A.M., A.M.) independently screened all retrieved records based on titles and abstracts. Studies were considered eligible if they addressed the clinical role of pediatric dentists and orthodontists in the management of children with OSA. Subsequently, full texts of potentially eligible articles were independently assessed by the same reviewers. Any discrepancies were resolved through discussion and, when necessary, consultation with a third reviewer (A.L.G.). The inclusion criteria were as follows: (1) studies involving pediatric populations (children and adolescents ≤ 18 years of age); (2) studies examining diagnostic, screening, or therapeutic aspects within the scope of pediatric dental or orthodontic practice; and (3) studies explicitly discussing the role, responsibilities, or clinical contributions of pediatric dentists and/or orthodontists in the multidisciplinary management of pediatric OSA. The exclusion criteria were (1) studies involving exclusively adult populations; (2) studies focusing solely on medical or surgical management; and (3) abstracts, editorials, or expert opinions without primary data. Data extraction and study selection were performed independently by two investigators (A.M., A.M.). In cases of disagreement, consensus was achieved through consultation with a third reviewer (A.L.G.). Extracted data were organized using a standardized data extraction form designed to categorize relevant study characteristics and outcomes, thereby facilitating structured narrative synthesis. The methodological quality of the included studies was assessed using the Scale for the Assessment of Narrative Review Articles (SANRA) [16]. Application of the SANRA criteria ensured a systematic appraisal of relevance, completeness, and scientific validity. Incorporating this tool into the review process enhanced the reliability of the findings and supported a structured synthesis of current evidence regarding the clinical responsibilities and practical tasks of pediatric dentists and orthodontists in the management of pediatric OSA. Appendix A presents the SANRA assessment of the present narrative review.

## 3. Results

From the initially identified 1536 records, 1134 remained after the exclusion of duplicates, and 968 records were excluded on the basis of title and abstract screening. A total of 166 full texts were assessed for eligibility, and 156 articles were excluded for not fulfilling the eligibility criteria. Thus, ten studies, all published in English, were identified as eligible and were included in the final qualitative synthesis (Table 2). A data extraction form was developed to categorize and list the information provided by the included studies in order to simplify the narrative elaboration of the present study. Important information was also retrieved from international guidelines, mentioned in the included studies (hand-searching), provided by leading scientific societies, i.e., the American Academy of Sleep Medicine (AASM) [17], American Academy of Dental Sleep Medicine (AADSM) [18], European Respiratory Society (ERS) [19], and American Association of Orthodontics (AAO) [20]. We also identified ten previously published reviews that argued about the critical role of pediatric dentists and orthodontists in the clinical management of children with OSA (Table 2). After examination of the full texts, the available evidence was filtered into specific clinical domains aimed at clarifying the role of the pediatric dentist and orthodontist in the management of pediatric obstructive sleep apnea (OSA). In particular, three main recommendations relevant to clinical practice were identified: the screening process, the diagnostic–therapeutic pathway and multidisciplinary management, and dental treatment options.

## 4. Discussion

### 4.1. Domain 1: Screening—The Role of the Pediatric Dentist and Orthodontist

The primary role of pediatric dentists and orthodontists in the screening of pediatric obstructive sleep apnea is the identification of clinical signs and symptoms within their scope of practice that may be associated with snoring and sleep-disordered breathing. Orthodontists, in particular, serve as effective diagnostic sentinels for several reasons: they routinely examine large pediatric populations beginning at approximately 5–6 years of age; they are familiar with multidisciplinary care pathways; and regular follow-up visits enable longitudinal observation and early detection of emerging concerns. Screening for pediatric snoring and OSA should integrate validated questionnaires with a comprehensive medical and dental history and a focused clinical examination. This assessment should emphasize both nocturnal and daytime symptoms, as reported by parents or caregivers, and should be conducted in accordance with current clinical recommendations [21,22].

#### 4.1.1. Signs and Symptoms

Children with OSA may present with nocturnal symptoms such as habitual snoring, which represents the most common clinical finding, witnessed apneas, choking or gasping episodes, and nocturnal enuresis. Parents may also report atypical sleep postures, including cervical hyperextension or sleeping with the head hanging off the bed, as well as restless sleep characterized by frequent positional changes. During daytime hours, some children exhibit excessive sleepiness, including the resumption of regular napping. More frequently, however, untreated OSA manifests as hyperactivity rather than somnolence, with behaviors resembling attention-deficit/hyperactivity disorder. These manifestations may include poor academic performance, inattention, morning headaches, irritability, and aggression. Although obesity represents a recognized risk factor for pediatric OSA, some affected children may instead present with failure to thrive. For this reason, systematic assessment of OSA risk should be incorporated into every comprehensive orthodontic evaluation [10,11,12].

#### 4.1.2. Validated Questionnaires

For initial screening, caregiver-completed questionnaires represent useful tools for identifying children at increased risk for OSA. However, they do not replace polysomnography (PSG), which remains the diagnostic gold standard. The Pediatric Sleep Questionnaire (PSQ) is a widely used parent-reported instrument designed to assess sleep-related breathing disorders and the main symptom clusters of pediatric OSA, including snoring, daytime sleepiness, and inattentive or hyperactive behavior [30,31]. It consists of a 22-item SRBD scale and three subscales (snoring, sleepiness, and behavior). Scores are calculated as the proportion of positive responses, with a commonly applied cutoff value of ≥0.33. The OSA-18 questionnaire evaluates health-related quality of life across five domains: sleep disturbance, physical symptoms, emotional symptoms, daytime functioning, and caregiver concerns. Total scores range from 18 to 126, with higher scores indicating a greater disease burden. Importantly, the OSA-18 primarily assesses disease impact rather than providing a direct diagnostic measure [30,31] and may therefore be particularly useful for longitudinal monitoring in dental settings. Both questionnaires may be administered through digital platforms, such as online forms or QR codes [30], allowing parents to complete them at home or in the waiting area of the dental clinic.

The Sleep Clinical Record (SCR) [32] represents a structured clinical scoring system that combines physical examination findings—such as tonsillar hypertrophy, nasal obstruction, and craniofacial characteristics—with symptom reporting. Originally developed and validated in pediatric populations, the SCR demonstrated improved diagnostic accuracy compared with symptom-based questionnaires alone in selected cohorts. However, its use requires a comprehensive upper airway assessment and specific clinical expertise in pediatric sleep-disordered breathing. Consequently, it is more frequently implemented in pediatric or otolaryngological settings than in routine dental practice.

The Brouillette OSA score, which is based on parental reports of snoring, witnessed apneas, and breathing difficulty during sleep, demonstrated reasonable predictive value in early validation studies [33]. Nevertheless, subsequent evidence has shown reduced sensitivity in mild OSA and limited ability to stratify disease severity. As a result, it has largely been replaced in clinical research by more comprehensive screening instruments, such as the Pediatric Sleep Questionnaire.

#### 4.1.3. Instrumental Screening Methods

In addition to questionnaires, instrumental screening methods are increasingly incorporated into pediatric OSA assessment. Overnight pulse oximetry currently represents the instrumental examination with diagnostic performance most closely approximating polysomnography in identifying apneic events. When clusters of oxygen desaturation are detected, pulse oximetry may suggest the presence of moderate-to-severe OSA. However, international guidelines emphasize that normal oximetry findings do not exclude the diagnosis, particularly in mild cases [19,34]. Meta-analytic evidence indicates that pulse oximetry demonstrates relatively high specificity but variable sensitivity in pediatric populations [31]. In pediatric dentistry and orthodontic settings, pulse oximetry may be considered when questionnaire results are positive or when craniofacial risk factors coexist, serving as an intermediate step prior to referral for polysomnography.

Portable home sleep apnea testing (HSAT) devices have received increasing attention. Nevertheless, the American Academy of Sleep Medicine does not recommend HSAT as a substitute for in-laboratory polysomnography in children, owing to insufficient validation and the risk of false-negative results [17]. Pediatric OSA is often characterized by subtle respiratory events and sleep fragmentation that may not be adequately detected by simplified home systems. Therefore, the use of HSAT in pediatric dental practice should remain cautious and be primarily limited to adjunctive screening purposes rather than definitive diagnosis.

In routine clinical practice, pediatric dentists and orthodontists may integrate these technologies into structured screening workflows. Pulse oximetry devices may be provided directly within the dental setting as an adjunctive service when questionnaire results are positive, or clinicians may recommend their use for home monitoring. Nevertheless, data obtained from questionnaires, instrumental screening tools, and orthodontic evaluation can only raise clinical suspicion and cannot establish a definitive diagnosis, which requires objective confirmation through polysomnography.

#### 4.1.4. Orthodontic Assessment

During screening, orthodontic evaluation should complement medical history and contribute to the identification of occlusal and craniofacial features associated with pediatric OSA, prompting referral to a sleep specialist when appropriate. Commonly reported findings include adenoid facies, maxillary constriction, mandibular retrusion with reduced chin projection (skeletal Class II), increased mandibular plane angle with a dolichofacial growth pattern, posterior crossbite, increased overjet, anterior open bite, lip incompetence, and dental crowding [35,36]. A meta-analysis by Liu et al. [37] confirmed a tendency toward skeletal Class II patterns associated with mandibular retrusion and facial hyperdivergency in children with OSA compared with controls. However, most cephalometric differences remained within normal diagnostic ranges. Therefore, these findings may have limited clinical relevance and should be interpreted with caution.

**Figure 1:**
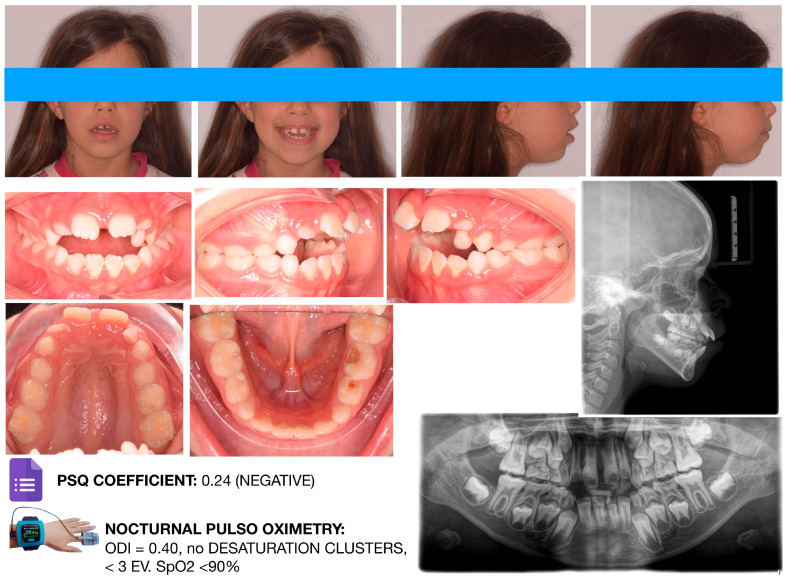
Female orthodontic patient of 8 y.o. featuring severe craniofacial characteristics compatible with an oral breathing pattern. Both screening tools, i.e., the PSQ and nocturnal pulse oximetry, provided negative results. Image courtesy of the authors.

**Figure 2:**
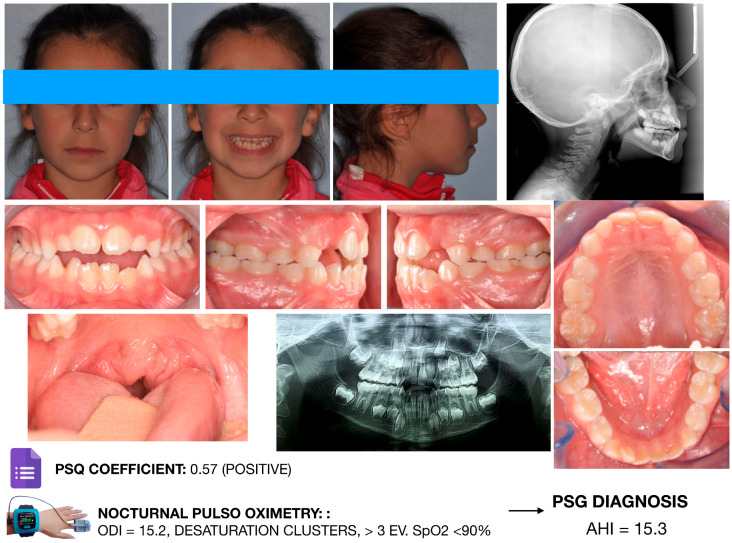
Female orthodontic patient of 8 y.o. featuring less severe craniofacial characteristics compatible with an oral breathing pattern. Both screening tools, i.e., the PSQ and nocturnal pulse oximetry, provided positive results. The patient also presented severe tonsillar hypertrophy. The patient was referred to the otorhinolaryngologist, and the diagnosis of sleep apnea was confirmed via PSG examination. Image courtesy of the authors.

Craniofacial features observed in children with OSA often overlap with those associated with chronic mouth breathing. In pediatric populations, in which adenotonsillar hypertrophy is prevalent, the association between mouth breathing and OSA appears stronger than in adults. Nevertheless, not all mouth breathers have OSA. Bokov et al. [38] reported that 41% of children with OSA were mouth breathers; notably, those without nasal obstruction often exhibited more severe and treatment-resistant disease. Consequently, craniofacial characteristics associated with the oral breathing phenotype may show limited sensitivity for identifying pediatric OSA. Figure 1 and Figure 2 illustrate two representative clinical cases supporting the concept that OSA cannot be diagnosed solely on the basis of occlusal or craniofacial features.

**Figure 3:**
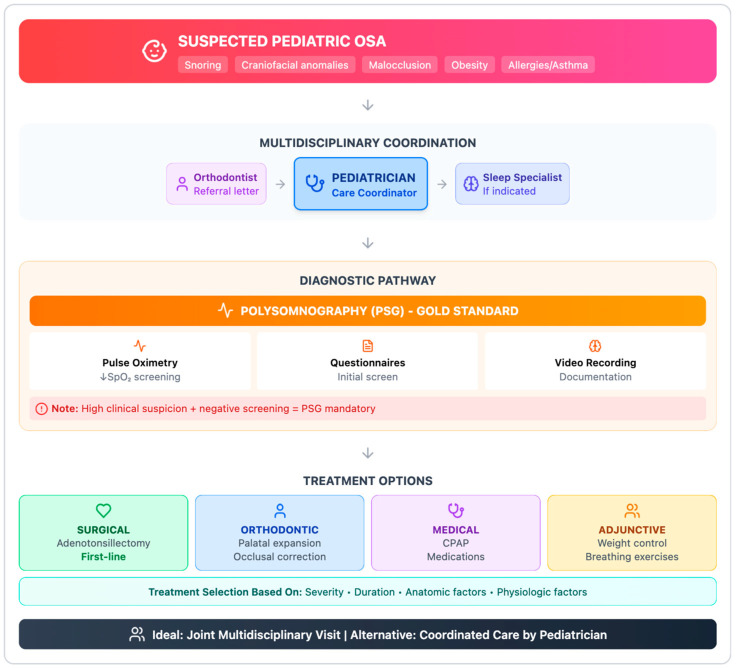
Roadmap for OSA management in pediatric patients.

Both patients exhibited orthodontic characteristics commonly reported in the literature, including maxillary constriction, anterior open bite, and a hyperdivergent facial pattern. However, although the patient shown in Figure 2 presented with a more complex orthodontic condition, both the Pediatric Sleep Questionnaire and overnight pulse oximetry yielded negative results. Conversely, the patient illustrated in Figure 3 displayed a less severe orthodontic phenotype; nevertheless, the PSQ was positive, and pulse oximetry revealed clusters of oxygen desaturation. The patient was therefore referred to an otolaryngologist and subsequently underwent in-laboratory polysomnography, which confirmed severe obstructive sleep apnea. In this case, apneic events were primarily attributable to severe adenoidal hypertrophy and tonsillar enlargement. These two cases provide a practical illustration of how pediatric dentists and orthodontists should structure the clinical screening workflow for pediatric patients at risk for OSA. Importantly, they highlight that all pediatric patients should be considered potentially at risk, regardless of their craniofacial or orthodontic characteristics. This process should include the following:

- A comprehensive clinical examination;
- Administration of validated screening questionnaires;
- Referral to an otolaryngologist or request for overnight pulse oximetry in the presence of a positive questionnaire (or doubtful parental anamnesis);
- Referral for polysomnography when pulse oximetry results are suggestive of sleep-disordered breathing.

### 4.2. Question 2: Diagnostic–Therapeutic Pathway and Multidisciplinary Management

The diagnostic and therapeutic pathway for growing patients with suspected snoring or obstructive sleep apnea should be inherently multidisciplinary. Children presenting with craniofacial or occlusal features associated with OSA, a history of habitual snoring, nasal obstruction, allergic rhinitis, asthma, obesity, or positive screening questionnaire results should be promptly referred to a pediatrician, who typically serves as the care coordinator. In this context, pediatric dentists and orthodontists should provide a structured referral letter summarizing relevant clinical findings and orthodontic assessments.

Because clinical evaluation alone is insufficient to establish a definitive diagnosis, in-laboratory polysomnography (PSG) is required to confirm the presence and severity of OSA [39]. When PSG is not readily available or feasible, alternative diagnostic tools, such as home video recordings or overnight pulse oximetry, may be considered preliminary assessments (Figure 3). However, these tools cannot replace polysomnography and should be interpreted within an integrated clinical framework.

In pediatric patients, oxygen desaturation frequently accompanies obstructive respiratory events. Therefore, reductions in peripheral oxygen saturation (SpO_2_) may indicate an increased likelihood of OSA and warrant further investigation with PSG. In this setting, pediatric dentists and orthodontists may contribute by providing pulse oximetry data as part of the referral documentation [40]. When clinical suspicion remains high despite negative screening or instrumental findings, referral to a sleep specialist and formal polysomnographic evaluation remain mandatory. Most children with confirmed OSA benefit from adenotonsillectomy and/or orthodontic treatment, depending on disease severity and underlying anatomical characteristics. Additional therapeutic options may include pharmacological management, weight control strategies, and continuous positive airway pressure (CPAP), particularly in patients with persistent symptoms, obesity, neuromuscular disorders, or complex craniofacial anomalies. Adjunctive breathing exercises and behavioral interventions may also provide supplementary benefits in selected cases.

Ideally, multidisciplinary assessment should occur during joint consultations involving pediatricians, otolaryngologists, sleep medicine specialists, and dental professionals. When this is not feasible, coordination of care remains primarily the responsibility of the pediatrician. Effective communication among healthcare providers is essential to ensure continuity of care, optimize treatment sequencing, and support long-term monitoring during growth and development. Within this multidisciplinary framework, pediatric dentists and orthodontists play a complementary role by facilitating early identification, supporting referral pathways, and contributing to post-treatment monitoring. Their ongoing clinical contact with pediatric patients places them in a strategic position to detect residual or recurrent symptoms and to promote timely re-evaluation when necessary.

### 4.3. Question 3: Therapeutic Options Related to Craniofacial Morphology

**Figure 4:**
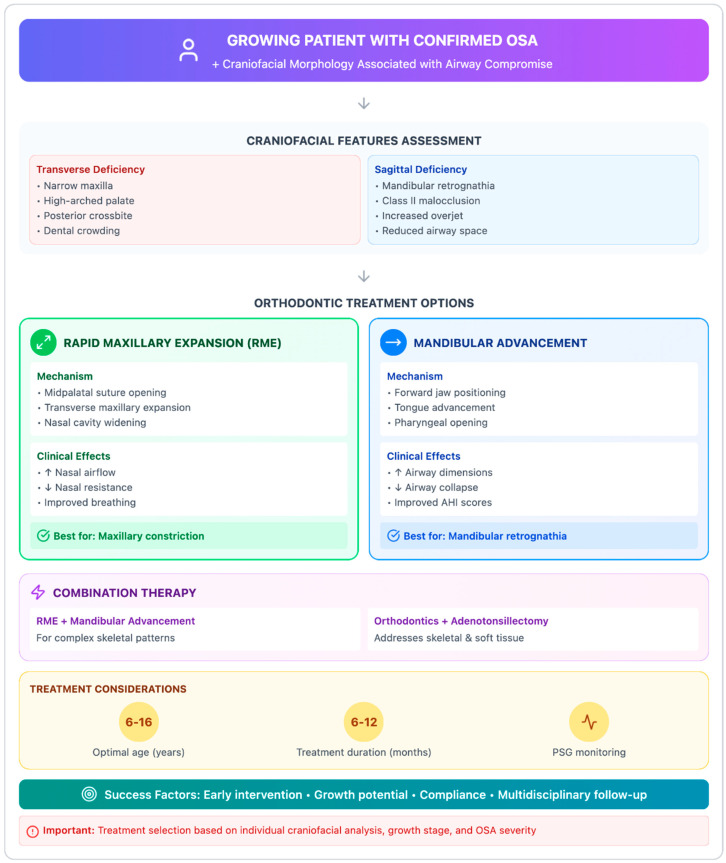
Treatment selection and considerations for pediatric OSA patients.

In growing patients with confirmed snoring or OSA and craniofacial morphology associated with airway compromise, orthodontic treatment options include rapid maxillary expansion (RME) and mandibular advancement appliances (Figure 4).

#### 4.3.1. Rapid Maxillary Expansion

Rapid maxillary expansion (RME) is an orthodontic intervention aimed at widening the maxillary arch, with direct effects on the nasal cavity and indirect effects on the upper airway [41,42]. A well-conducted meta-analysis [43] reported an average reduction of approximately 3.24 events/hour in the apnea-hypopnea index (AHI) following RME, with most included studies showing post-treatment AHI values approaching the normal range. Two main mechanisms have been hypothesized to explain the potential respiratory benefits of RME in pediatric OSA: (1) transverse expansion of the posterior maxilla may increase nasopharyngeal volume by laterally displacing the surrounding soft tissues [44], and (2) the documented reduced nasal airway resistance and improved airflow dynamics after expansion may decrease inspiratory negative pressure, thereby reducing pharyngeal collapsibility [45]. Both mechanisms may secondarily reduce chronic lymphoid tissue irritation (low certainty of evidence), potentially exerting a favorable effect in pediatric OSA, where adenotonsillar hypertrophy represents the primary etiological factor [19].

However, many studies included in the meta-analysis and several subsequent investigations present significant methodological limitations that substantially restrict the ability to draw definitive conclusions regarding the interpretation of treatment effects. These limitations include the absence of untreated control groups and the lack of stratification according to age or skeletal maturation stage. Consequently, reported improvements in AHI should be interpreted with caution, as airway modifications may reflect not only treatment effects but also physiological growth processes. In particular, spontaneous regression of adenotonsillar tissue represents a major confounding factor in pediatric populations. Adenoidal tissue, as part of Waldeyer’s ring, typically increases in size until approximately 5–7 years of age and subsequently undergoes physiological involution. This developmental pattern overlaps with the peak incidence of pediatric OSA and adenotonsillar hypertrophy [46,47]. As a result, respiratory improvements observed within 6–12 months of follow-up cannot be confidently attributed exclusively to maxillary expansion. Supporting this interpretation, recent computational fluid dynamics (CFD) studies comparing children aged 6–9 years with those aged 11–14 years have reported greater nasopharyngeal airflow changes in younger patients. However, these differences are likely influenced by age-related regression of lymphoid tissues rather than solely by expansion-induced skeletal modifications [34,48,49,50].

When translated into clinical practice, these findings suggest that patient age represents a predominant determinant of respiratory improvement following RME, given the central role of adenotonsillar hypertrophy in pediatric OSA pathophysiology. In cases of persistent or severe non-regressing adenotonsillar hypertrophy, surgical management remains the primary therapeutic approach. Current evidence does not support orthodontic treatment as a first-line therapy for pediatric OSA [20]. Accordingly, palatal expansion should be performed exclusively in the presence of clear orthodontic indications, such as transverse maxillary deficiency and/or posterior crossbite. Any associated respiratory improvement should be regarded as a potential secondary benefit rather than a primary treatment objective. Nevertheless, orthodontic intervention may play a supportive role in selected patients with residual OSA after adenotonsillectomy, particularly when craniofacial constriction contributes to persistent airway compromise. Consistent with this cautious approach, the literature supports a watchful waiting strategy in children with mild sleep-disordered breathing, as spontaneous enlargement of the upper airway following physiological regression of adenotonsillar tissue may lead to symptom improvement in up to 50% of untreated cases [15]. Otolaryngologists are therefore encouraged to reserve surgical intervention for severe or persistent airway obstruction. Similarly, pediatric dentists and orthodontists should initiate orthodontic treatment only when clear dentofacial indications are present, while remaining aware of potential airway-related effects.

With regard to craniofacial morphology, current evidence does not demonstrate that specific anatomical phenotypes reliably predict greater AHI reduction following RME. However, structural features such as nasal septum deviation, mandibular retrusion, and inferior turbinate hypertrophy have been associated with partial relapse after adenotonsillectomy. Patients presenting with Mallampati grade III–IV in combination with reduced mandibular dimensions and a narrow nasomaxillary complex may therefore exhibit a limited response to surgery and could represent candidates for adjunctive orthodontic evaluation in the management of residual OSA [15].

**Figure 5:**
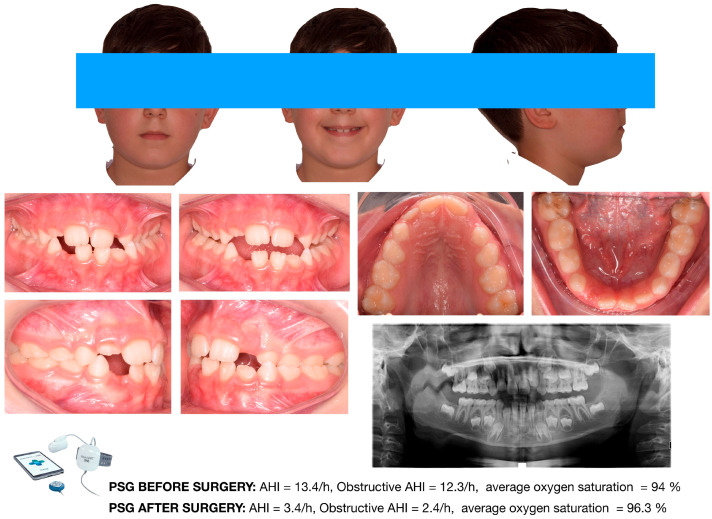
Male orthodontic patient of 8 y.o., referred by the ORL specialist, presenting functional posterior cross-bite and requiring maxillary expansion. The ORL specialist planned adenotonsillectomy before performing maxillary expansion. After adenotonsillectomy, the AHI dropped to 3.4/h, the obstructive AHI dropped to 2.4/h, and the average oxygen saturation increased to 96.3%. Image courtesy of the authors.

**Figure 6:**
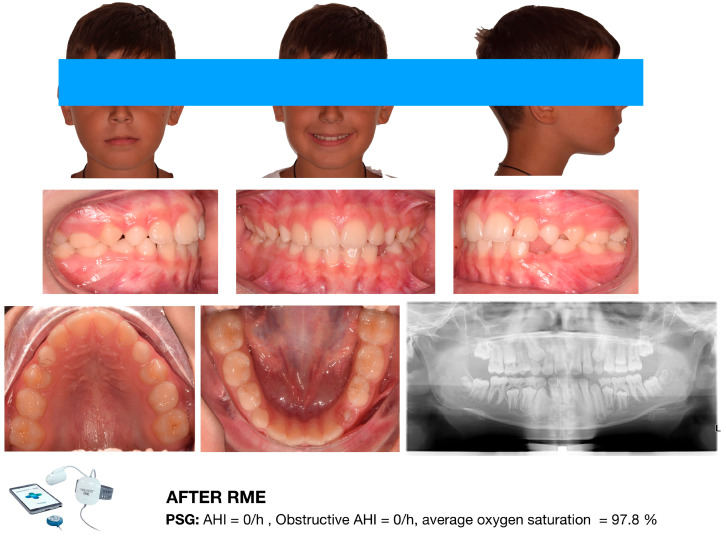
Same male patient after RME. Residual obstructive apnea events were eliminated immediately after maxillary expansion. Image courtesy of the authors.

Figure 5 and Figure 6 illustrate the case of an 8-year-old male patient who underwent RME for correction of a functional posterior crossbite following adenotonsillectomy. After completion of the active expansion phase, residual AHI values were normalized. However, such outcomes must be interpreted within a multidisciplinary framework, as improvements may reflect both treatment-induced anatomical changes and physiological growth processes.

Long-term follow-up studies and well-controlled prospective trials remain necessary to establish standardized clinical protocols and evidence-based recommendations. Future research should stratify patients according to age, craniofacial pattern, soft-tissue status, and baseline OSA severity in order to clarify if and which pediatric phenotype may derive the greatest benefit from maxillary expansion.

#### 4.3.2. Mandibular Advancement Appliances

Mandibular advancement aims to enlarge the retrolingual airway space by anteriorly repositioning the mandible and tongue in patients with obstructive sleep apnea. In adult populations, mandibular advancement devices are primarily used to achieve positional airway stabilization. In contrast, in children and adolescents, functional appliances are mainly intended to stimulate mandibular growth and modify skeletal development rather than to provide positional advancement alone. A recent meta-analysis reported reductions in the apnea-hypopnea index (AHI) following treatment with mandibular advancement appliances. However, substantial heterogeneity among included studies was observed, and the overall certainty of evidence was rated as low to very low. Moreover, inconsistent effects on oxygen saturation parameters were reported. Interpretation of these findings is further limited by the frequent absence of untreated control groups and by the confounding influence of normal craniofacial growth. These methodological limitations are comparable to those identified in studies evaluating the effects of rapid maxillary expansion in pediatric OSA patients. Consequently, current evidence does not support the use of mandibular advancement appliances as stand-alone therapy for pediatric OSA in the absence of clear orthodontic indications [20].

**Figure 7:**
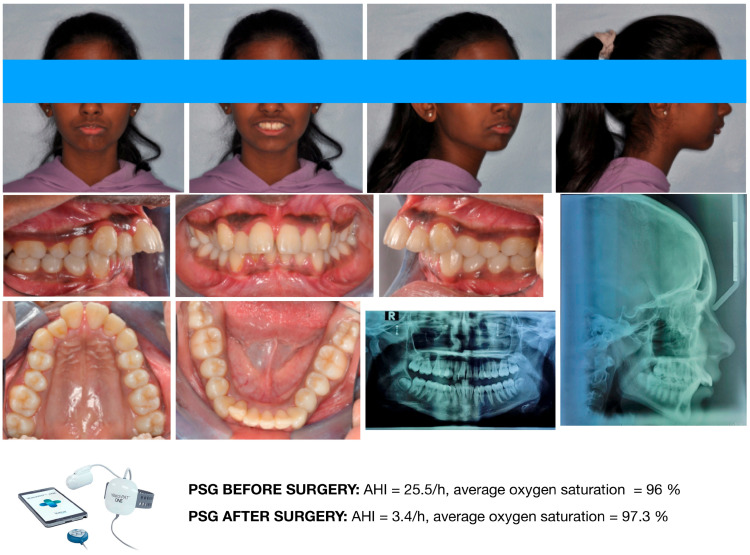
Female orthodontic patient of 12 y.o., referred by the ORL specialist, presenting severe Class II malocclusion with mandibular retrusion. The ORL specialist planned adenotonsillectomy before orthodontic treatment. After adenotonsillectomy, the AHI dropped to 3.4/h, and the average oxygen saturation increased to 96.3%. Image courtesy of the authors.

**Figure 8:**
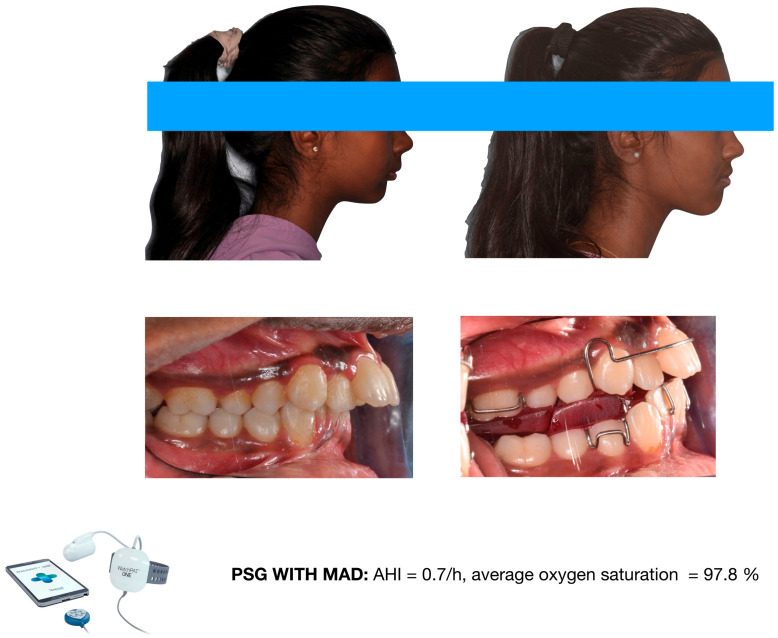
The same female patient during treatment with a functional appliance (mandibular advancement device). Residual obstructive apnea events were eliminated while wearing the appliance. Image courtesy of the authors.

Accordingly, mandibular advancement devices should primarily be prescribed for orthodontic purposes, such as the management of mandibular retrusion or skeletal Class II malocclusion. Their use as adjunctive interventions may be considered in carefully selected patients. One potential clinical indication involves growing patients with mandibular retrusion who present with residual OSA following delayed adenotonsillectomy, provided that treatment is initiated during an appropriate growth phase for functional orthopedic intervention. In such cases, mandibular advancement appliances may contribute to the reduction in residual respiratory events while simultaneously addressing underlying dentofacial disharmony (Figure 7 and Figure 8). Nevertheless, treatment planning should remain embedded within a multidisciplinary framework, and therapeutic outcomes should be interpreted cautiously in light of ongoing skeletal maturation.

#### 4.3.3. Orofacial Myofunctional Therapy

Orofacial myofunctional therapy (MFT) has emerged as a potential adjunctive intervention in the multidisciplinary management of pediatric obstructive sleep apnea. MFT consists of structured exercises designed to improve the tone, coordination, and endurance of the oropharyngeal and perioral musculature, with the objective of enhancing upper airway stability during sleep. The pathophysiological rationale for MFT is based on the concept that impaired neuromuscular control of the upper airway contributes to airway collapsibility. In pediatric patients, factors such as muscular hypotonia, chronic mouth breathing, and altered tongue posture may further compromise airway patency, particularly in the presence of craniofacial constriction or adenotonsillar hypertrophy. By promoting nasal breathing, appropriate tongue positioning against the palate, and improved lip seal, MFT aims to reduce upper airway resistance and support functional craniofacial development. Systematic evidence suggests that myofunctional therapy may reduce the apnea–hypopnea index (AHI) and improve oxygen saturation parameters. However, most available data derive from heterogeneous populations and relatively small cohorts. A systematic review and meta-analysis by Camacho et al. [51] reported significant reductions in AHI in both adults and children undergoing oropharyngeal exercises, with greater effects observed when therapy was used as an adjunct rather than as a stand-alone intervention. In pediatric populations, Guilleminault et al. [52] demonstrated that MFT following adenotonsillectomy reduced the risk of residual OSA, highlighting its potential role in preventing disease recurrence. Similarly, Villa et al. [53] reported improvements in respiratory parameters and symptom scores in children who adhered to structured myofunctional protocols.

Despite these encouraging findings, current international guidelines do not recognize MFT as a first-line therapy for pediatric OSA. Nevertheless, they acknowledge its potential role as a complementary intervention, particularly in cases of residual OSA after adenotonsillectomy or in children with persistent mouth breathing and orofacial dysfunction [19]. From a clinical perspective, MFT may be particularly indicated in children presenting with the following:

- Chronic oral breathing despite adequate nasal patency;
- Low tongue posture or anterior tongue thrust;
- Lip incompetence;
- Mild residual OSA after primary surgical management;
- Neuromuscular hypotonia contributing to airway instability.

Treatment success appears to depend strongly on patient adherence and family engagement, and standardized pediatric protocols remain insufficiently defined. High-quality randomized controlled trials with long-term follow-up are therefore required to clarify the optimal timing, duration, and integration of MFT within orthodontic and surgical treatment pathways. Accordingly, pediatric dentists and orthodontists may consider incorporating myofunctional therapy into comprehensive, individualized management plans, particularly when functional orofacial dysfunction coexists with structural airway risk factors [24].

### 4.4. Future Research Directions

Significant gaps persist in the evidence supporting the role of orthodontic and adjunctive therapies in pediatric obstructive sleep apnea (OSA). A key priority concerns the assessment of the long-term stability of orthodontic interventions—particularly rapid maxillary expansion (RME) and mandibular advancement—since current literature predominantly documents short- to medium-term improvements in the apnea-hypopnea index (AHI). Well-designed prospective longitudinal studies extending through adolescence are warranted to evaluate the durability of respiratory outcomes, their interaction with craniofacial growth, and potential relapse rates.

Future research should also prioritize the identification of phenotypic predictors of treatment response. Pediatric OSA represents a heterogeneous disorder, influenced by craniofacial morphology, adenotonsillar hypertrophy, obesity, and neuromuscular tone. Stratified approaches integrating anatomical and functional phenotyping may enhance patient selection and optimize the timing of orthodontic intervention within multidisciplinary treatment protocols, including surgical and medical management. Randomized controlled trials assessing combined and sequential treatment strategies—such as adenotonsillectomy followed by orthodontic or myofunctional therapy—are also needed to define evidence-based therapeutic pathways during growth and development.

Emerging technologies—such as wearable sleep monitoring systems, automated signal processing, and machine learning-based pattern recognition—offer promising tools for early diagnosis and longitudinal follow-up. Moreover, investigational research on inflammatory, genetic, and neuromuscular biomarkers may improve risk stratification. However, these approaches require validation in large, multicenter cohorts before translation into routine clinical practice.

## 5. Conclusions

Pediatric obstructive sleep apnea (OSA) is a multifactorial disorder that requires early recognition and coordinated, multidisciplinary management to prevent long-term adverse health consequences. On the basis of the current evidence, clinical recommendations for pediatric dentists and orthodontists may be structured around three principal domains.

Screening: Pediatric dentists and orthodontists play a pivotal role as frontline sentinels in the identification of children at risk for OSA. Routine dental and orthodontic appointments provide a strategic opportunity to detect suggestive signs and symptoms, administer validated screening questionnaires, and recognize craniofacial and occlusal characteristics associated with increased airway vulnerability. Although these findings are not diagnostic per se, they enable timely referral for definitive assessment, including polysomnography when indicated. From a practical standpoint, the integration of QR code–based questionnaires into the clinical workflow and the use or prescription of instrumental screening tools—such as nocturnal pulse oximetry—may enhance early detection and streamline referral pathways.

Diagnostic–therapeutic pathway and multidisciplinary management: Close collaboration with pediatricians, otolaryngologists, and sleep medicine specialists is essential. Within a multidisciplinary framework, dentists and orthodontists contribute by providing structured referrals, documenting relevant clinical findings, and sharing adjunctive screening data when appropriate. Such coordination facilitates accurate diagnosis, supports patient monitoring (including watchful waiting strategies), and optimizes the timing and sequencing of interventions during growth. In clinical practice, this may involve drafting formal referral letters to pediatricians, otolaryngologists, and speech therapists that clearly communicate (a) positive questionnaire or instrumental screening findings and/or (b) the orthodontic needs of children considered at risk for OSA.

Dental and oral treatment options: Orthodontic and adjunctive interventions—including rapid maxillary expansion, mandibular advancement devices, and orofacial myofunctional therapy—may provide supportive benefits in carefully selected patients. However, current evidence does not support their use as stand-alone treatments for pediatric OSA. Orthodontic therapy should be undertaken primarily for established dentofacial indications (e.g., transverse maxillary deficiency or skeletal Class II relationships), with potential airway-related improvements regarded as secondary outcomes. Treatment effects must be interpreted with caution, given the confounding influence of growth and developmental changes. Clinically, ongoing multidisciplinary communication is recommended to coordinate decisions regarding (a) the indication for adenotonsillectomy or watchful waiting, (b) post-surgical outcomes assessed by polysomnography or pulse oximetry, and (c) post-orthodontic follow-up, including evaluation for residual OSA.

In summary, pediatric dentists and orthodontists occupy a critical position within the interdisciplinary management of pediatric OSA. Their contribution to early screening, structured referral, and appropriately indicated orthodontic intervention may enhance comprehensive care, provided that treatment decisions remain evidence-based and integrated within a coordinated clinical pathway.

## Figures and Tables

**Table 1:** Consulted databases, search strategies, and the number of retrieved articles.

Databases	Search Strategy Used	Results
**MEDLINE** searched via PubMed www.ncbi.nlm.nih.gov/sites/entrez/(accessed on 1 November 2025)	(((Sleep apnoea syndrome *) OR (exp Sleep Apnoea, Obstructive)) OR (Upper airway resistance sleep apnoea syndrome*)) OR (OSA OR OSAS)) OR (SNORING OR PRIMARY SNORING)) AND ((children) OR (growing subjects)) AND ((dentist *) OR (pediatric dentist *) OR (pedodontist *) OR (orthodontist *) OR (orthodontics) OR (orthodontic treatment)))	847
**WEB OF SCIENCE** www.webofknowledge.com (accessed on 1 November 2025)	TS = ((“sleep apnoea syndrome *” OR “obstructive sleep apnoea *” OR “upper airway resistance sleep apnoea syndrome*” OR OSA OR OSAS OR snoring OR “primary snoring”) AND (children OR “growing subjects”) AND (dentist * OR “pediatric dentist *” OR pedodontist * OR orthodontist * OR orthodontics OR orthodontic treatment))	260
**SCOPUS** www.scopus.com (accessed on 1 November 2025)	ALL (sleep apnoea syndrome OR obstructive sleep apnoea OR upper airway resistance sleep apnoea syndrome OR OSA OR OSAS OR snoring OR primary snoring) AND ALL (children OR growing subjects) ALL (dentist OR “pediatric dentist” OR pedodontist OR orthodontist OR orthodontics OR orthodontic treatment)	429
TOTAL		1536

**Table 2:** Studies included for the purpose of the present narrative review.

Authors	Years	Journal	Title
Mallappa, D.R.J.S. [21]	2025	International Journal of Applied Dental Sciences	Obstructive Sleep Apnea: A multidisciplinary challenge: The roles of pediatric dentists, orthodontists, and prosthodontists in diagnosis and management: A comprehensive review
Fagundes, N.C.F. [15]	2022	Pediatric Pulmonology	Pediatric obstructive sleep apnea: Dental professionals can play a crucial role
Heit, T. [22]	2022	Children MDPI (Basel)	Craniofacial Sleep Medicine: The Important Role of Dental Providers in Detecting and Treating Sleep Disordered Breathing in Children
Giuca, M.R. [23]	2021	Scientific World Journal.	Pediatric Obstructive Sleep Apnea Syndrome: Emerging Evidence and Treatment Approach
Moin Answer, H.M. [24]	2021	Saudi Dental Journal	The role of the dentist in the diagnosis and management of pediatric obstructive sleep apnea
Luzzi, V. [25]	2019	zEuropean Review for Medical and Pharmacological Sciences	Obstructive sleep apnea syndrome in the pediatric age: the role of the dentist
Behrents, R.G. [20]	2019	American Journal of Orthodontics and Dentofacial Orthopedics	Obstructive sleep apnea and orthodontics: An American Association of Orthodontists White Paper
Stark, T.R. [26]	2018	Sleep Medicine Clinics	Pediatric Considerations for Dental Sleep Medicine
Leibovitz, S. [27]	2017	Quintessence International	Pediatric sleep-disordered breathing: Role of the dentist
Padmanabhan, V. [28]	2010	Journal of Clinical Pediatric Dentistry	Sleep disordered breathing in children—a review and the role of a pediatric dentist
Tzur-Gadassi, L. [29]	2014	Refuat Hapeh Vehashinayim	Pediatric obstructive sleep apnea—an orthodontic perspective

## Data Availability

The data presented in this study are available on request from the corresponding author due to privacy restrictions.

## Appendix A. SANRA Criteria of the Present Review

**Sanra Criteria**	**Score**	**Scoring Reason**
1. Importance for the readership	2	The introduction contains all the elements to explain the significance of the communication deficits of individuals.
2. Stated aims or research questions	2	The aims of the study are stated clearly in the abstract and Section x with main research questions and subobjectives.
3. Description of the literature search	2	A transparent, structured search is described. The databases, keywords, Boolean, date range, inclusion/exclusion criteria, and the use of Kable’s 12-step method are noted.
4. Referencing	2	2 Up-to-date, relevant, and peer-reviewed references were used from databases.
5. Scientific reasoning	2	The discussion was developed by synthesizing the findings across all studies and supported with citations and any possible evidence.
6. Appropriate presentation of data	2	The results were described by generating three domains according to the emerging recommendation available in the literature.
